# Molecular identification and phylogenetic characterization of *Leptospira* spp. in domestic animals from Jordan

**DOI:** 10.5455/javar.2026.m1044

**Published:** 2026-06-22

**Authors:** Mohammad H. Gharaibeh, Sawsan S. Jarrah, Sameeh M. Abutarbush, Farah R. Al Qudsi, Ibrahim M. Alzuheir

**Affiliations:** 1 Department of Basic Veterinary Medical Science, Faculty of Veterinary Medicine, Jordan University of Science and Technology, P.O. Box 3030, Irbid 22110, Jordan; 2 Faculty of Graduate Studies, Jordan University of Science and Technology, P.O. Box 3030, Irbid 22110, Jordan; 3 Department of Clinical Veterinary Medical Science, Faculty of Veterinary Medicine, Jordan University of Science and Technology, P.O. Box 3030, Irbid 22110, Jordan; 4 Department of Nutrition and Food Technology, Jordan University of Science and Technology, Irbid 21121, Jordan

**Keywords:** *Leptospira* spp., molecular characterization, phylogenetic analysis, domestic animals, One Health

## Abstract

**Background:** Leptospirosis is an emerging zoonotic disease-causing substantial morbidity, mortality, and economic losses worldwide, with over 60,000 human deaths annually. Early diagnosis and molecular identification of *Leptospira* spp. are essential for effective control and prevention. In Jordan, while serological evidence exists, there is a critical lack of molecular data regarding the species circulating in animal hosts.

**Objectives:** This study aimed to detect *Leptospira* infection using nested polymerase chain reaction (PCR) and to characterize the isolates molecularly through sequencing and phylogenetic analysis of the *16S rRNA* (*rrs*) gene.

**Materials and Methods:** DNA was extracted from serum and urine samples collected between January 2020 and May 2021 from 82 animals suspected of leptospirosis, including 80 cattle, one goat, and one dog. Pooled urine from imported feedlot calves and urine from the goat and dog were analyzed. Positive *Leptospira* samples were identified using nested PCR targeting the *rrs* gene, followed by Sanger sequencing. Phylogenetic analysis was performed using MEGA software, and sequences were compared with reference serovars from GenBank.

**Results:** Three samples (3.5%) were positive for the *Leptospira* *rrs* gene, producing the expected 299 bp amplicon. Sequencing confirmed *Leptospira* DNA in two samples—one from pooled urine of imported calves and one from a dog serum sample. The sequences were submitted to GenBank as “Uncultured *Leptospira* spp. Canine Jordan 2020” (OK394051) and “Uncultured *Leptospira* spp. Cattle Jordan 2020” (OK394045).

**Conclusions:** This is the first molecular characterization of *Leptospira* spp. in domestic animals in Jordan. The findings underscore the need to include leptospirosis in differential diagnoses of febrile diseases and to enhance surveillance and preventive programs.

## 1. Introduction

Leptospirosis is a widespread yet neglected zoonotic disease that poses a substantial threat to human, animal, and environmental health globally [1]. As a classic “One Health” challenge, its transmission cycle involves a complex interplay between various hosts—including livestock, rodents, companion animals, and wildlife—and the environments they share [2]. The disease is caused by pathogenic spirochetes of the genus *Leptospira*, a genetically diverse group comprising three major evolutionary lineages and more than 300 serovars [3]. While historical records of the disease in the Middle East date back to the early 20th century, it remains a re-emerging global concern, particularly as climate change and unplanned urbanization create conditions favorable for bacterial survival and transmission [4, 5]. The public health and economic impacts of *Leptospira* are profound. In humans, infection can manifest as a broad clinical spectrum, from a mild flu-like illness to life-threatening Weil’s disease, characterized by multi-organ failure and pulmonary hemorrhage [6]. The pathogen is maintained in nature through the chronic renal colonization of asymptomatic reservoir hosts, primarily rodents, which shed the bacteria via urine into soil and water [7]. Domestic animals, such as cattle, dogs, and pigs, act as critical amplifying hosts, facilitating transmission to humans through direct contact or environmental contamination during agricultural, occupational, or recreational activities [8].

Diagnosing leptospirosis remains a significant challenge due to the non-specific nature of its clinical presentation and the difficulty in differentiating pathogenic from saprophytic strains using conventional phenotypic methods [9]. While serological assays like the Microscopic Agglutination Test (MAT) are the diagnostic gold standard, they are limited by a delayed antibody response—often undetectable until 7–10 days post-infection—and cross-reactivity between serovars [10]. In contrast, molecular techniques such as polymerase chain reaction (PCR) offer high sensitivity and specificity for early-stage detection during the leptospiremic phase [11]. Nested PCR assays targeting conserved genes, such as the *16S rRNA* (*rrs*) gene, or pathogen-specific markers like *lipL32*, have emerged as robust tools for identifying and phylogenetically classifying *Leptospira* species [12].

In Jordan, the epidemiological landscape of leptospirosis is poorly defined. A 2019 serological survey in dairy cattle identified a high prevalence of serovars Pomona and Hardjo, pointing toward environmental risk factors such as contaminated water sources [13]. However, the absence of molecular data has prevented the identification of the actual species involved and their genetic relationships with regional or global strains. Given the high density of livestock and the zoonotic risk at the human-animal interface, molecular surveillance is essential for developing targeted control strategies. Therefore, this study aimed to detect and molecularly characterize *Leptospira* spp. in Jordanian domestic animals using nested PCR and phylogenetic analysis, providing a foundational step toward a comprehensive One Health surveillance framework in the region.

## 2. Materials and Methods

### 2.1. Ethics approval

The study protocol was reviewed and approved by the Institutional Animal Care and Use Committee (IACUC) at Jordan University of Science and Technology (JUST), Irbid, Jordan (Project # 163/2015). All sample collection was completed in compliance with national and institutional animal welfare guidelines.

### 2.2. Sample collection

**Figure 1.**
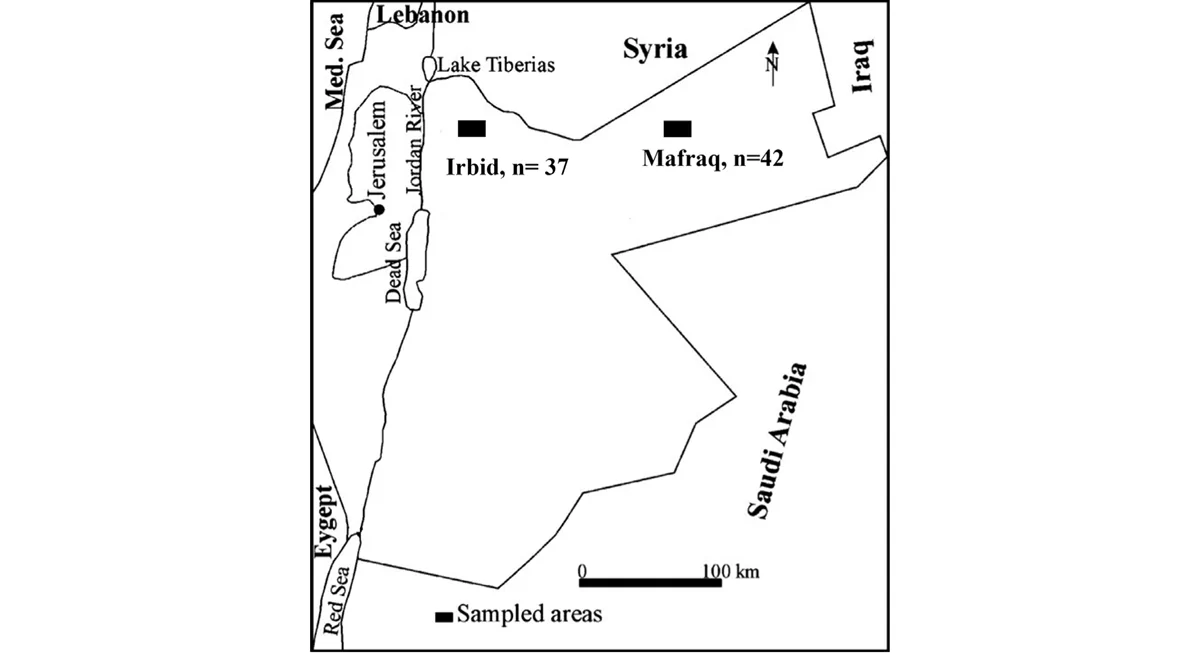
**Map of Jordan showing the geographic locations of the sampling areas included in this study.** Archived serum samples from dairy cows were collected from farms in Irbid (*n* = 37) and Mafraq (*n* = 42) governorates during 2019. All molecular analyses were conducted at the Jordan University of Science and Technology (JUST) laboratory.

This study was conducted from January 2020 to May 2021 at the Jordan University of Science and Technology (JUST) laboratory. The study analyzed a total of 85 samples, consisting of 82 serum samples and 3 urine samples. The sample set was composed of 79 archived serum samples from dairy cows collected in 2019 from farms located in Irbid (*n* = 37) and Mafraq (*n* = 42) governorates, one archived serum sample from a calf collected in 2016, and prospective blood and urine samples collected from one goat, one dog, and a pooled urine sample from a group of imported feedlot calves from Hungaria. All investigated animals showed hematuria (Figure 1).

Whole blood (approximately 10 ml) was collected via jugular venipuncture into plain vacutainer tubes, with serum separated by centrifugation at 3000× *g* for 12 min and stored at –20°C. Urine samples were collected in sterile containers and subjected to centrifugation at 3500× *g* for 12 min, with the resulting pellets harvested and stored at –20°C for subsequent analysis.

### 2.3. DNA extraction and nested PCR

DNA was extracted from 400 µl of urine pellets and serum using the DNeasy Blood and Tissue Kit (Qiagen, Germantown, MD, USA) following the manufacturer’s instructions for animal cells. Urine pellets were processed according to Qiagen’s protocol (Cat. No. 69504) with minor modifications as described by Djadid et al. [14]. DNA was eluted in 200 µl of buffer AE and stored at –20°C until use for molecular detection by nested PCR. The nested PCR assay was employed to detect *Leptospira* DNA by targeting a 299 bp fragment of the *16S rRNA* (*rrs*) gene.

The first-round 50 µl reaction mixture contained 5 µl of template DNA, 10 µM each of primers Forward (5′–GGC GGC GCG TCT TAA ACA TG–3′) and Reverse (5′–TTC CCC CCA TTG AGC AAG ATT–3′), 1× HotStart Taq Master Mix (Qiagen), and Q-Solution. Thermal cycling included initial activation at 95°C for 15 min, followed by 30 cycles of 94°C for 1 min, 63°C for 1 min, and 72°C for 1 min, with a final extension at 72°C for 10 min. The second-round reaction used 5 µl of the first-round product as a template with nested primers Forward (5′–TGC AAG TCA AGC GGA GTA GC–3′) and Reverse (5′–TTC TTA ACT GCT GCC TCC CG–3′) under identical cycling parameters. Amplicons were resolved by electrophoresis on a 1.5% agarose gel stained with ethidium bromide and visualized under UV illumination, utilizing *Leptospira bataviae* DNA and RNase-free water as positive and negative controls, respectively.

### 2.4. Sequencing and phylogenetic analysis of the rrs gene

Positive amplicons of the expected size (299 bp) were excised from the gel, purified, and subjected to bidirectional Sanger sequencing by Macrogen (Seoul, South Korea). The resulting sequences were edited and analyzed using Sequencing Analysis Software v5.3.1 (Applied Biosystems), and sequence identity was confirmed through BLASTn analysis against the NCBI GenBank database. Phylogenetic reconstruction was conducted using the Neighbor-Joining method. The bootstrap consensus tree inferred from 1000 replicates is taken to represent the evolutionary history of the taxa analyzed. Branches corresponding to partitions reproduced in less than 70% bootstrap replicates are collapsed. The percentage of replicate trees in which the associated taxa clustered together in the bootstrap test (1000 replicates) is shown next to the branches. The evolutionary distances were computed using the maximum composite likelihood method and are in the units of the number of base substitutions per site. This analysis involved 28 nucleotide sequences. Codon positions included were 1st + 2nd + 3rd + Noncoding. All ambiguous positions were removed for each sequence pair (pairwise deletion option). There were a total of 1519 positions in the final dataset. Evolutionary analyses were conducted in MEGA X [15].

## 3. Results

### 3.1. Molecular detection of Leptospira DNA

**Figure 2.**
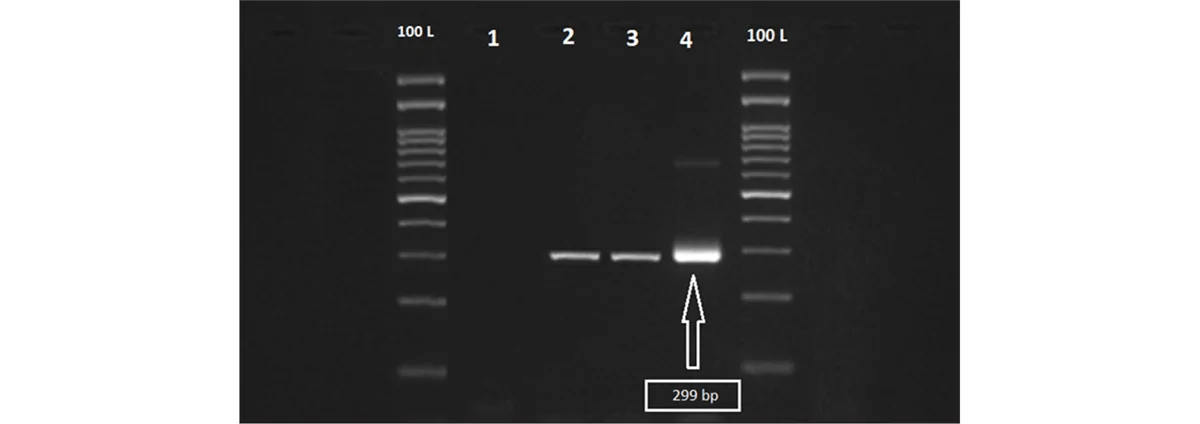
**Gel electrophoresis analysis using a 1.5% agarose gel for detection of the *16S RNA* gene (299 bp).** Lane 100 L is a GeneDireX^®^ (Taoyuan, Taiwan); Lane 1 is a negative control, 4 is a positive control, and 2–3 are *Leptospira* positive samples.

A total of 85 samples (82 serum and 3 urine) collected from domestic animals in Jordan were screened for *Leptospira* spp. using a nested PCR assay targeting the *16S rRNA* (*rrs*) gene. Analysis revealed an overall positivity rate of 3.5% (3/85). Specifically, the *rrs* gene was detected in 2.4% (2/82) (95% CI: 0.7–8.5%) of serum samples and 33.3% (1/3) (95% CI: 6.1–79.2) of urine samples. The positive amplicons exhibited the expected band size of 299 bp (Figure 2). The positive samples included one serum sample from a Jordanian calf, one serum sample from a dog, and one pooled urine sample from a group of imported feedlot calves. Detailed nested PCR results are summarized in Table 1.

**Table 1.** Nested PCR results of 85 serum and urine samples from suspected animals.

PCR results	Serum n (%) [95% CI]	Urine n (%) [95% CI]	Total samples n (%) [95% CI]
Positive	2 (2.4%) [0.7–8.5]	1 (33.3%) [6.1–79.2]	3 (3.5%) [1.2–9.9]
Negative	80 (97.6%) [91.5–99.3]	2 (66.7%) [20.8–93.9]	82 (96.5%) [90.1–98.8]
Total	82 (100%)	3 (100%)	85 (100%)

### 3.2. Sequencing and BLAST analysis

Bidirectional Sanger sequencing was performed on the three PCR-positive amplicons. The sequence from the Jordanian calf serum sample was excluded from further analysis due to high background noise in the chromatogram. The remaining two sequences were successfully analyzed and deposited in GenBank under the following names: “Uncultured *Leptospira* spp. Canine Jordan 2020” (OK394051) and “Uncultured *Leptospira* spp. Cattle Jordan 2020” (OK394045).

BLASTn analysis of the cattle-derived sequence (OK394045) revealed 98% identity with *Leptospira borgpetersenii* serovar Hardjo (U12670) and *Leptospira interrogans* strain IP1507003 (MH329312.1). The canine-derived sequence (OK394051) showed 97.4% similarity to *Leptospira kirschneri* strain 201001687 (JN683874.1). Sequence similarity between the two Jordanian strains was 94%.

### 3.3. Phylogenetic characterization

**Figure 3.**
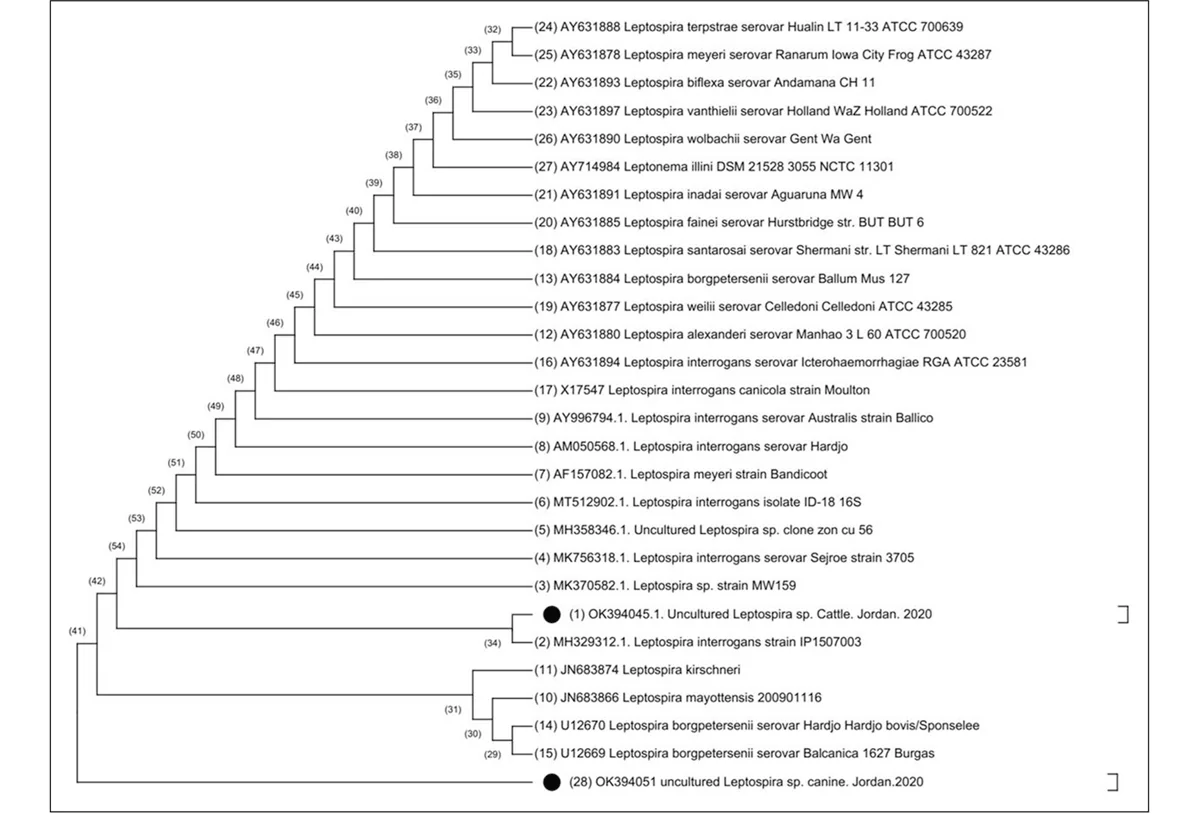
**Phylogenetic relationship of Jordanian *Leptospira* isolates (OK394051 and OK394045) based on the partial *16S rRNA* (*rrs*) gene sequence.** The evolutionary history was inferred using the Neighbor-Joining method. The bootstrap consensus tree inferred from 1000 replicates is taken to represent the evolutionary history of the taxa analyzed. Branches corresponding to partitions reproduced in less than 70% bootstrap replicates are collapsed. The percentage of replicate trees in which the associated taxa clustered together in the bootstrap test (1000 replicates) is shown next to the branches. The evolutionary distances were computed using the maximum composite likelihood method and are in the units of the number of base substitutions per site. Evolutionary analyses were conducted in MEGA X [4]. The Jordanian strains are nested within the pathogenic clade, showing close evolutionary proximity to *L. interrogans*, *L. kirschneri*, and *L. borgpetersenii*.

Phylogenetic analysis was conducted to determine the evolutionary relationship between the Jordanian strains and international reference strains. The resulting neighbor-joining tree (Figure 3) demonstrated that both Jordanian sequences belong to the same major clade of pathogenic *Leptospira*. The cattle isolate (OK394045) was genetically clustered with *L. interrogans* strain IP1507003 (MH329312.1), while the canine isolate (OK394051) clustered closely with *L. kirschneri* (JN683874.1), *L. borgpetersenii* serovar *Hardjo* (U12670), and *L. mayottensis* (JN683866).

## 4. Discussion

Leptospirosis is a globally distributed yet frequently neglected zoonotic disease [16]. In the Middle East, particularly in Jordan, epidemiological data regarding the circulating species and their molecular characteristics have been critically sparse [17]. Prior investigations in Jordan primarily utilized serological methods, reporting a high seroprevalence of 26.2% in healthy dairy cattle [13]. While previous studies have reported the molecular detection of Leptospira in Jordanian livestock [18], the present study represents the first targeted molecular characterization and phylogenetic analysis of *Leptospira* spp. in clinically suspected domestic animals and archived specimens within the country, contributing to an increased dataset for molecular screening of *Leptospira* spp.

Our findings revealed a molecular positivity rate of 3.5% (3/85) using nested PCR. This rate is notably lower than the reported seroprevalence in the region, a discrepancy also observed in studies from Egypt, where PCR positivity was as low as 1.1% in asymptomatic herds [19]. Such variations are likely due to the transient nature of leptospiremia and the timing of sample collection relative to the infection stage. Within the first 10 days of infection, the pathogen is typically detectable in the blood; subsequently, it migrates to the kidneys and is intermittently shed in the urine [20, 21]. In our study, the dog tested positive in serum but negative in urine, suggesting an early-stage infection prior to the onset of leptospiruria.

The detection of *Leptospira* DNA in the pooled urine of imported feedlot calves is a finding of significant biosecurity concern. These calves, arriving from Hungary, presented with clinical symptoms seven days post-arrival. Given the typical incubation period of 2–20 days and the fact that urinary shedding begins approximately 10 days post-infection, it is plausible that these animals were infected either at the point of origin or during transit. This underscores the risk of transboundary animal diseases and emphasizes the necessity of rigorous molecular screening of imported livestock, as recommended by the World Health Organization [22].

Phylogenetic analysis based on the *rrs* gene provided high-resolution insights into the pathogenic lineages circulating in Jordan. The cattle-derived sequence (OK394045) exhibited 98% identity with *L. borgpetersenii* serovar Hardjo and *L. interrogans* strain IP1507003, which are globally recognized causes of bovine reproductive failure and has been previously suspected in Jordan based on MAT results [13]. Its identification in this study reinforces its status as a major regional veterinary and public health threat, especially given recent outbreaks in neighboring territories [23]. The canine isolate (OK394051) showed high similarity (97.4%) to *L. kirschneri*, a species frequently associated with canine leptospirosis and human infection in Southeast Asia and parts of Africa [24, 25]. Interestingly, this isolate also shared 96% similarity with a rat-derived strain from Egypt, suggesting a possible common environmental reservoir or cross-species transmission cycle in the region. However, the epidemiological significance of this similarity cannot be determined from the current dataset and would require additional environmental and wildlife sampling.

While the *rrs* gene is an excellent target for screening due to its presence across the genus, its limited taxonomic resolution often necessitates the use of nested PCR to enhance sensitivity. Our study utilized nested PCR to detect loads as low as 20 bacterial cells/ml [26]. However, we found that even with high sensitivity, sequencing can be hampered by background noise or low DNA concentration, as seen in our excluded bovine serum sample. This aligns with findings by Esteves et al. [27], where PCR-positive samples occasionally fail to yield high-quality sequences. Future studies should incorporate additional markers such as *lipL32*, *secY*, or multilocus sequence typing (MLST) to improve species-level resolution.

The economic burden of leptospirosis in Jordan is exacerbated by delayed diagnosis and a lack of clinical awareness. Because symptoms mimic other febrile and icteric diseases, the infection often goes untreated, leading to increased transmission and severe complications [28, 29]. Furthermore, the overcrowding and suboptimal sanitation observed on some Jordanian dairy farms facilitate the environmental persistence of *Leptospira*.

## 5. Conclusions

In conclusion, this study establishes the first molecular footprint of pathogenic *Leptospira* species in Jordanian livestock and companion animals. The identification of *L. interrogans* and *L. kirschneri* lineages highlights the zoonotic risk at the human-animal-environment interface. To mitigate this risk, leptospirosis must be integrated into the differential diagnosis protocols for febrile animals and humans in Jordan. Furthermore, the detection of pathogens in imported calves calls for a standardized “One Health” surveillance program and more stringent quarantine measures for transboundary animal movements to prevent the introduction of novel pathogenic serovars.

## Data Availability

The datasets generated and analyzed during the current study are available in the NCBI GenBank repository under accession numbers OK394045 (“Uncultured *Leptospira* spp. Cattle Jordan 2020”) and OK394051 (“Uncultured *Leptospira* spp. Canine Jordan 2020”).

## List of abbreviations

BLASTn, Basic Local Alignment Search Tool for nucleotides; bp, base pair; DNA, deoxyribonucleic acid; JUST, Jordan University of Science and Technology; MAT, Microscopic Agglutination Test; MEGA, Molecular Evolutionary Genetics Analysis software; NJ, Neighbor-Joining; PCR, polymerase chain reaction; *rrs*, *16S ribosomal RNA* gene; RNA, ribonucleic acid; UV, ultraviolet; WHO, World Health Organization; min, minute; ml, milliliter; µl, microliter.
